# Supplementary material for: AAV2 bypasses direct endosomal escape by using AAVR to access the trans-Golgi network en route to the nucleus
Source: J Virol. 2026 Jul 29;100(8):e00337-26. doi: 10.1128/jvi.00337-26 (PMC13483459; doi:10.1128/jvi.00337-26)
Supplement: Supplementary file 1 [file jvi.00337-26-s0001.docx]

**Supplementary Figures**

**Supplementary Figure 1.**

Transmission electron microscopy (TEM) of liposomes incubated with individual or pairwise components. Neither retromer, SNX3, nor the AAVR cytosolic tail peptide (residues 956–974) alone, nor any two-component combination, is sufficient to induce tubulation. Robust tubule formation requires the concerted presence of retromer, SNX3, and a cargo peptide.

**Supplementary Figure 2. Quantification of retromer-SNX3-mediated liposome tubulation by DMT1-II and AAVR cargo peptides.** **(a)** Percentage of tubulated liposomes following overnight incubation with retromer, SNX3, and the indicated cargo peptide: DMT1-II (550–568), AAVR (956–974), or AAVR-Δ (956–967, lacking the predicted retromer-binding motif). Bars show mean ± SD of n = 3 independent experiments; individual replicates shown as dots. **(b)** Total tube length per liposome (nm) for the same conditions; mean ± SD, n = 3. **(c)** Distribution of individual tube lengths measured from TEM images for DMT1-II (n = 35 tubes) and AAVR (n = 27 tubes). Violin plots show median (circle), mean (diamond), and interquartile range (bar). AAVR-Δ omitted from **(c)** as no tubulation was observed.

**Supplementary Figure 3.**

Western Blot of purified wild-type AAV2 and AAV2-ΔVP1 capsid preparations with anti-AAV VP1/VP2/VP3 B1 antibody (ARP, 690058). Wild-type AAV2 shows the expected VP1, VP2, and VP3 bands, while the AAV2-ΔVP1 preparation lacks VP1 and retains VP2 and VP3, confirming the absence of VP1 in the mutant capsids.

**Supplementary Figure 4.**

Proximity ligation assay (PLA) for EEA1 and intact AAV2 capsids (A20) in naïve HuH-7 (left) and AAVR-knockout HuH-7 cells (right) at 6 hours post-transduction. PLA signal (red) indicates close proximity (<40 nm) between AAV2 particles and the early endosomal marker EEA1. AAVR-KO cells display markedly stronger PLA signal compared to naïve HuH-7 cells, indicating that in the absence of AAVR, AAV2 particles accumulate in early endosomal compartments rather than progressing to the TGN via retrograde transport. DAPI (blue) labels nuclei. Scale bars: 50 µm.
